# Label-free detection of real-time DNA amplification using a nanofluidic diffraction grating

**DOI:** 10.1038/srep31642

**Published:** 2016-08-17

**Authors:** Takao Yasui, Kensuke Ogawa, Noritada Kaji, Mats Nilsson, Taiga Ajiri, Manabu Tokeshi, Yasuhiro Horiike, Yoshinobu Baba

**Affiliations:** 1 Department of Applied Chemistry, Graduate School of Engineering, Nagoya University, Furo-cho, Chikusa-ku, Nagoya 464-8603, Japan; 2 ImPACT Research Center for Advanced Nanobiodevices, Nagoya University, Furo-cho, Chikusa-ku, Nagoya 464-8603, Japan; 3 Japan Science and Technology Agency (JST), PRESTO, 4-1-8 Honcho, Kawaguchi, Saitama 332-0012, Japan; 4 Science for Life Laboratory, Department of Biochemistry and Biophysics, Stockholm University, Se-171 21 Solna, Sweden; 5 Graduate School of Chemical Sciences and Engineering, Hokkaido University, Sapporo 060-8628, Japan; 6 Division of Applied Chemistry, Faculty of Engineering, Hokkaido University, Sapporo 060-8628, Japan; 7 National Institute for Materials Science, Tsukuba 305-0044, Japan; 8 Health Research Institute, National Institute of Advanced Industrial Science and Technology (AIST), Takamatsu 761-0395, Japan

## Abstract

Quantitative DNA amplification using fluorescence labeling has played an important role in the recent, rapid progress of basic medical and molecular biological research. Here we report a label-free detection of real-time DNA amplification using a nanofluidic diffraction grating. Our detection system observed intensity changes during DNA amplification of diffracted light derived from the passage of a laser beam through nanochannels embedded in a microchannel. Numerical simulations revealed that the diffracted light intensity change in the nanofluidic diffraction grating was attributed to the change of refractive index. We showed the first case reported to date for label-free detection of real-time DNA amplification, such as specific DNA sequences from *tubercle bacilli* (TB) and human papillomavirus (HPV). Since our developed system allows quantification of the initial concentration of amplified DNA molecules ranging from 1 fM to 1 pM, we expect that it will offer a new strategy for developing fundamental techniques of medical applications.

Amplifying DNA molecules quantitatively, such as using a polymerase chain reaction (PCR)^1^ or rolling circle amplification (RCA)^2^, not only provides insights into basic medical and molecular biological research but also serves as a fundamental technique for diagnostic and medical applications. Monitoring real-time DNA amplification using fluorescence labeling in a PCR manner has been successfully used to quantify DNA amplification; however this PCR-based method could potentially bias quantification including the following: amplification^3^ and fluorescence-reagent volume^4^. PCR-based amplification issue occurs due to PCR errors (sequence artifacts) and unequal or cloning efficiency, and fluorescence-reagent volume issue occurs due to pipetting process. One way to avoid the issues related to the PCR-based method is employing circle-to-circle amplification (C2CA), which is known to have less amplification issue for the monitoring real-time DNA amplification^5^,^6^; however this C2CA-based method could also potentially bias quantification due to hybridization^7^. For the monitoring real-time DNA amplification, C2CA-based method still needs fluorescent detection using oligonucleotide-tagged fluorophores^5^, leading to hybridization issue.

Here we report label-free detection of real-time DNA amplification in a C2CA manner, which has less amplification, fluorescence-reagent volume, and hybridization issues. C2CA-based method is well suited to overcoming the amplification issues^5^,^6^, and label-free detection is well suited to overcoming the fluorescence-reagent volume and hybridization issues. A number of research groups have contributed to development of such label-free detection methods as plasmon sensing^8^, nanowire sensing^9^, microcantilever sensing^10^, and whispering gallery mode sensing^11^ methods. Although these methods can achieve high sensitivity but they require the target molecules are immobilized on them usually using specific molecules to capture the targeted ones. Such surface immobilization of specific molecules before use is a major procedure, being: labor- and time-intensive, expensive to perform, incompatible with some materials, and performance-impaired over time. For a capture of DNA molecules, researchers generally use complementary probe oligonucleotides immobilized on the surface, potentially introducing hybridization bias. Therefore, none of these methods have achieved truly label-free real-time monitoring of DNA amplification. Here we propose a label-free detection method with no surface immobilization, which utilizes detection of intensity changes of diffracted light produced by a nanofluidic diffraction grating during DNA amplification in free solution.

Our strategy for quantifying DNA amplification in a label-free manner is based on observations of intensity changes of diffracted light derived from the passage of a laser beam through nanochannels embedded in a microchannel; the ability to diffract the beam is a basic characteristic of nanochannels (Fig. 1). The use of nanochannels for label-free DNA detection has been reported by several groups^12^,^13^,^14^, however to the best of our knowledge, their use is potentially expected to detect electrical signals during DNA translocation, and therefore, their use is not appropriate to real-time monitoring of DNA amplification for a long time. For the real-time monitoring of DNA amplification, the fabricated nanochannels (Fig. 1a,b) are 800 nm periodic nanogrooves (200 nm wide grooves, which we used as nanochannels) with 2.7 μm depth, embedded in the microchannel, and they were fabricated on fused silica substrates by electron beam lithography, photolithography, and plasma etching (Supplementary Fig. S1), as described elsewhere^15^. For an incident laser beam in the label-free detection system, we used a modulated 532 nm laser to amplify a specific component extracted from random or background noise (Fig. 1c). The 532 nm laser beam was focused by an objective lens and was incident to and diffracted by the nanofluidic diffraction grating (Fig. 1d). A two-dimensional (2D) intensity profile of the diffracted light is shown in Fig. 1e. A photodiode with a 100 μm aperture pinhole was used for a detector and a detected signal was fed into a lock-in-amplifier. We manipulated the position of the photodiode to get local maximum signals in the diffracted light, and got sufficiently large signal changes. Specific signal changes were derived from target molecules (e.g. various liquids and DNA molecules) passing through the nanofluidic diffraction grating or DNA amplification in it, and monitored in the data logger.

Since our developed system detects a change in the intensity of the diffracted light during DNA amplification into the 200 nm wide grooves in the nanofluidic diffraction grating, we achieve a label-free detection without any surface immobilization for a long time; with the detection, we are working toward the goal of combining label-free detection and real-time DNA amplification in a single chip. The ability to diffract the laser beam, which the nanochannels has intrinsically, is governed by the diffraction condition expressed as *pn*_*1*_(sin*θ*_*0*_ + sin*θ*_*1*_) = *mλ*, where *p* is the pitch (800 nm), *n*_*1*_ is the refractive index of fused silica substrate (1.46), *θ*_*0*_ is an incident angle (90°), *θ*_*1*_ is a diffractive angle, *m* is an integer number, and *λ* is the wavelength of the incident laser (532 nm). The diffracted angle *θ*_*1*_ values of 27.1° and 65.6° were calculated from the equations for the first order and the second order, respectively. These diffracted light beams are refracted at the interface between the fused silica substrate and the atmosphere, and the refraction angle follows Snell’s law; *n*_*1*_sin*θ*_*1*_ = *n*_*2*_sin*θ*_*2*_. Considering the refractive index in the atmosphere is 1.00, we calculated the refraction angle *θ*_*2*_ values of 41.7° and >90° for the first order and the second order, respectively; the diffracted light of the first order was visible at 41.7° and the diffracted light of the second order was total-reflected (Fig. 1f). We measured the transmissivity for the 532 nm wavelength at each output angle and confirmed that the 532 nm laser beam had some transmittance at 42° (Fig. 1g). This experimental point showed good agreement with the refraction angle for the diffracted light of the first order as calculated above.

First we carried out label-free detection by introducing various liquids into the 200 nm wide grooves in the nanofluidic diffraction grating to verify the detection ability of our system based on the intensity change of the diffracted light. When various liquids were introduced into the 200 nm wide grooves, the refractive index inside the grooves changed; hence, the difference in the refractive index of between the 200 nm wide grooves (depending on the various liquids used as sample) and fused silica substrate (1.46) changed. For a proof-of-concept, we monitored intensity changes, normalized ∆I (∆I was defined as the intensity difference from the initial state after sample introduction), introducing water molecules as shown in Fig. 2a,b; the refractive index in the nanochannels changed from air to water molecules. Before introduction of water (Fig. 2c), we detected label-free signals for the air condition, and then, as we gradually filled the nanochannels embedded in the microchannel with water by using capillary force (Fig. 2d), the intensity of the diffracted light gradually decreased, and finally the intensity reached a plateau when the nanochannels were filled (Fig. 2b). 2D intensity profiles of the diffracted light in Fig. 2e,f revealed that the total signal intensity decreased after the introduction of water. Intensity changes, normalized ∆I, introducing several liquids in response to the refractive indices of them in Fig. 2g showed that our detection mechanism is primarily based on the change of refractive index. Refractive indices of the various liquids were measured with a high-precision refractometer and their refractive indices were calculated; these values are summarized in Supplementary Table S1. As the refractive indices were close to the refractive index for fused silica substrate, diffraction efficiency would decrease, resulting in an increase for the intensity change of the diffracted light. On the other hand, the bigger the difference between the refractive indices of the various liquids and the refractive index for fused silica substrate, the smaller the intensity changes of the diffracted light.

Numerical simulations using rigorous coupled wave analysis (RCWA) revealed that the change of the refractive indices by introducing various liquids into the 200 nm wide grooves in the nanofluidic diffraction grating could trigger the intensity change of the diffracted light (Fig. 2h). A decrease of the diffraction efficiency would be accompanied by an intensity decrease of diffracted light, resulting in an increase of normalized intensity changes. The diffraction efficiency decreased as the refractive indices of the various liquids were close to the refractive index for fused silica substrate. These RCWA simulated results showed good agreement with the experimental points (Fig. 2g,h). We concluded that the label-free signals were produced from the change of the refractive indices in the nanochannels; the label-free signals were the intensity changes attributed to the change of the diffraction efficiency introducing various liquids. Our label-free detection system based on the change of the refractive index of the sample could also recognize different molecular compositions in a solution by exchanging solutions using electroosmotic flow as described in Supplementary Fig. S2; *e.g.*, from water molecules to tris-borate-EDTA molecules in water or from tris-borate EDTA molecules in water to tris-EDTA molecules in water. The intensity change of the diffracted light when introducing DNA molecules into the 200 nm wide grooves in the nanofluidic diffraction grating showed that our label-free detection system could achieve good sensitivity for label-free detection of DNA molecules (Supplementary Fig. S3). Coupled with the label-free detection of DNA molecules (non-absorbing molecules at 532 nm), we used our developed system to detect absorbing molecules, which have light absorption at 532 nm, in a label-free manner (Supplementary Fig. S4).

Our developed system could achieve label-free detection of real-time DNA amplification in the nanofluidic diffraction grating based on the intensity change of the diffracted light (Fig. 3). Our amplification scheme is based on circle-to-circle amplification (C2CA) (Supplementary Fig. S5 and Supplementary Table S3)^5^. Phi29 DNA polymerase can be used to amplify DNA molecules in the C2CA reaction^16^,^17^. To detect the amplified DNA molecules in a real-time label-free manner we set the room temperature at 34 °C (Supplementary Fig. S6). The nanofluidic diffraction grating was warmed up uniformly for this room temperature (Supplementary Fig. S7). The RCA reaction is initiated following introduction of ligated product into the 200 nm wide grooves in the nanofluidic diffraction grating (Fig. 3a). Because the RCA product could influence the intensity change of the diffracted light, we achieved the label-free detection of real-time DNA amplification (Fig. 3b). When compared to the intensity change for the negative control, we could conclude that the isothermal process at 34 °C did not influence the intensity changes, normalized ∆I, during DNA amplification.

Figure 3b shows the first case reported to date for label-free detection of real-time DNA amplification, which amplified specific sequences derived from *tubercle bacilli* (TB) and human papillomavirus (HPV). To the best of our knowledge, only one experimental report has performed refractive index detection using nanochannels^18^, however, the detection sensitivity is not sufficient for label-free detection of real-time DNA amplification (10^−3^ refractive index units (RIU)). Since our developed system could achieve over 10^−5^ RIU detection sensitivity (Supplementary Fig. S3 and Supplementary Table S2), our developed system could detect the intensity change of the diffracted light amplifying DNA molecules. And also, all types of DNA sequences would be detectable in our developed system whenever the RCA reaction is initiated. The RCA reaction for real-time DNA amplification of TB sequence was confirmed using a molecular beacon (Supplementary Fig. S8). We note that the real-time label-free detection of the RCA reaction showed linear amplification in the same way as fluorescent detection of the RCA reaction using the molecular beacon, and it was previously shown that the highly processive phi29 DNA polymerase can synthesize a DNA strand at the rate of at least 1000 nt/min in a linear fashion^19^. Comparison with the negative control (no target sequence) revealed our developed system could recognize the label-free signal derived from the RCA reaction with an initial concentration of 1 pM within at least 2.5 minutes, which gave a signal at 3 SDs (standard deviations) above the background.

The changes of refractive index were responsible for the intensity change of the diffracted light when amplifying DNA molecules in the nanofluidic diffraction grating. We measured refractive index during the DNA amplification for the TB sequence in response to amplification time with the high-precision refractometer, and the values are summarized in Table S4. The refractive index during the DNA amplification clearly changed with the amplification time (Fig. 3c). Using those refractive indices and the RCWA method, we simulated diffraction efficiency during the DNA amplification. It showed good correspondence with the experimental points (Fig. 3d). A decrease of diffraction efficiency during the DNA amplification occurred with an accompanying intensity decrease of diffracted light, resulting in an increase of normalized ∆I. We concluded that the label-free detection of real-time DNA amplification in the nanofluidic diffraction grating was achieved by the change of the refractive index during the DNA amplification in the device; the label-free signals of real-time DNA amplification were the real-time intensity changes attributed to the change of the diffraction efficiency amplifying DNA molecules. Our results might suggest the use of commercially available refractometers to perform label-free detection of real-time DNA amplification. However, considering that refractometers typically require sample volumes ranging from a couple of hundreds μL to mL, we believe that our technique, which supplies only 1 μL to sample reservoirs, is more compatible with most biological sample volumes.

The changes of refractive index derived from the DNA amplification remind us of the changes of refractive index seen for increasing length of polymer chains. In polymer physics, glass-transition temperature, density, and refractive index for longer polymer chains are much higher than those for shorter ones due to the liquids in the free-space^20^, even if the longer polymer chains are polymerized from the shorter polymer chains in bulk; both longer and shorter polymer chains have the same composition. DNA amplification in the nanofluidic diffraction grating has the same condition as polymer chains, *i.e.*, the composition of sample in the nanofluidic diffraction grating during DNA amplification differs little before and after DNA amplification. From the perspective of polymer physics, it makes sense that the refractive index for DNA molecules with the same composition can vary during DNA amplification.

Our developed system allowed us to quantify the initial concentration of amplified DNA molecules ranging from 1 fM to 1 pM (Fig. 3e,f), which is difficult to detect using fluorescence-based detection system (100 pM; Supplementary Fig. S8 and the reported data by Dahl, F. *et al.*^5^). Figure 3e shows label-free detection of real-time DNA amplification with different initial concentrations for the TB sequence, as normalized ∆I versus amplification time in wide range of concentrations; from 1 fM to 1 pM. Time-course monitoring of normalized ∆I in the wide range of concentrations indicated a linear increase similar to the linear trend of the RCA reaction^19^. Comparison with the negative control (no target sequence) revealed our developed system could recognize the label-free signal derived from the RCA reaction for TB sequence with an initial concentration of 1 pM, 100 fM, 10 fM, and 1 fM for 2, 4, 6.5, and 14 minutes, respectively, which gave a signal at 3 SDs above the background. A plot for the label-free detection of real-time DNA amplification as normalized ∆I at 15 minutes versus logarithmic scale initial concentration of the target sequence provided a limit of detection of 500 aM, which gave a signal at 3 SDs above the background (Fig. 3f). Thus these experimental data prove that our developed system can well quantify the initial concentration of target sequence in a wide concentration range (1 fM-1 pM) in a label-free manner, which is not achieved using fluorescence-based methods (detection limit: 100 pM), *e.g.*, Supplementary Fig. S8 and the reported data^5^. Since the present novel concept based on the nanochannels embedded in a microchannel has the potential to realize label-free detection of real-time DNA amplification and label-free quantification of the initial concentration of target sequence with lower concentration range rather than fluorescence-based methods, this will open up new research avenues for not only detecting real-time DNA amplification but also developing novel analytical methods for various biological molecules, which are not attainable by existing methods.

In summary, we have demonstrated a novel label-free detection method using the nanofluidic diffraction grating, which enables detection of real-time DNA amplification for a wide range of initial concentrations (1 fM-1 pM), quantitatively. Our developed system could determine the concentration of target molecules from calibration curves, even though our target molecules were absorbing and non-absorbing molecules. The label-free detection method using the nanofluidic diffraction grating will play an important role in achieving much better resolution in quantitative DNA amplification. Furthermore, our developed system could amplify DNA at relatively low temperature (34 °C) with no thermal cycles. Since the described results strongly indicate that our label-free detection system has potential applicability to simultaneous label-free detection and real-time DNA amplification in a single chip, we propose this system is a new strategy for molecular biological research and fundamental medical applications, and for developing an inexpensive miniaturized system of bacteria and viruses detection.

## Methods

### Label-free detection system

The incident laser beam was the 532 nm emission line of a diode pumped solid-state laser (GCL-075-S, Luminex Trading, Inc.) with 75 mW output power. The incident laser beam was passed through an ND filter (VND-50U, Sigma Koki Co., Ltd.) and modulated by a light chopper (5584A, NF Electronic Instruments Inc.) with a modulation frequency at 1013 Hz. The modulated laser beam was focused by a 10×/0.30 NA objective lens (Olympus Corp.) and diffracted by the nanochannels when the laser beam was incident perpendicular to the nanofluidic diffraction grating. The diffracted light was detected with a photodiode (ET-2030, Japan Laser Corp.) that had a 100 μm aperture pinhole (PA-100, Sigma Koki Co., Ltd.) and was fed into a lock-in-amplifier (LI5640, NF Electronic Instruments Inc.). The digitized signal was output through a data logger (TR-V500, Keyence Corp.). The time constant of the lock-in amplifier was set to 100 ms, and the sampling rate of the data logger used was 10 ms for the detection of various liquids or DNA molecules, and for the detection of real-time DNA amplification, the time constant of the lock-in amplifier and the sampling rate of the data logger used were each set to 30 s.

### 2D intensity profile for the diffracted light

The 2D images of the intensity profile for the diffracted light were captured with a digital CCD camera (C8484-05G02, Hamamatsu Photonics K.K.) with fiber optic plate (FOP) screen (A6502, Hamamatsu Photonics K.K.), and the detected profile was analyzed by LEPAS-12 (C9334-01, Hamamatsu Photonics K.K.).

### Measurement of transmissivity

The transmissivity of 532 nm at each output angle was measured with an automated absolute reflectance measurement system (ARMV-734, Jasco Corp.). Wavelength ranging from 300 to 800 nm was used for incident light. Incident angle of the incident light was set to 0° and transmissivity of the output angle was measured from 0 to 90°.

### Calculation of refractive index at 532 nm

The refractive index was measured with a high-precision refractometer (KPR-2000, Shimadzu Corp., Kyoto, Japan) at room temperature with 50% humidity (minimum volume: 2 mL). The refractive index at 532 nm wavelength was calculated using the five-term Herzberger equation (1):


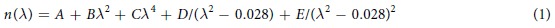


where *A*, *B*, *C*, *D*, and *E* are approximation coefficients, *n*(*λ*) is an refractive index at an arbitrary wavelength, and *λ* is the arbitrary wavelength. *A*, *B*, *C*, *D*, and *E* are solutions to a system of equations from five combinations of wavelength and refractive index.

### Simulations using RCWA method

Rigorous coupled wave analysis (RCWA; Diffracted MOD, RSoft Design Group Japan KK, Tokyo, Japan) was used for simulation of diffraction efficiency. In the RCWA method, the wavelength, background refractive index, and difference of the refractive index can be set. The model was two-dimensional and grid size was 0.1 nm. In our system, the wavelength was 532 nm and the background refractive index was that of fused silica 1.46071; we could simulate diffraction efficiency from the refractive index of the target various liquids^21^.

## Additional Information

**How to cite this article**: Yasui, T. *et al.* Label-free detection of real-time DNA amplification using a nanofluidic diffraction grating. *Sci. Rep.* **6**, 31642; doi: 10.1038/srep31642 (2016).

## Supplementary Material

Supplementary Information

## Figures and Tables

**Figure 1:**
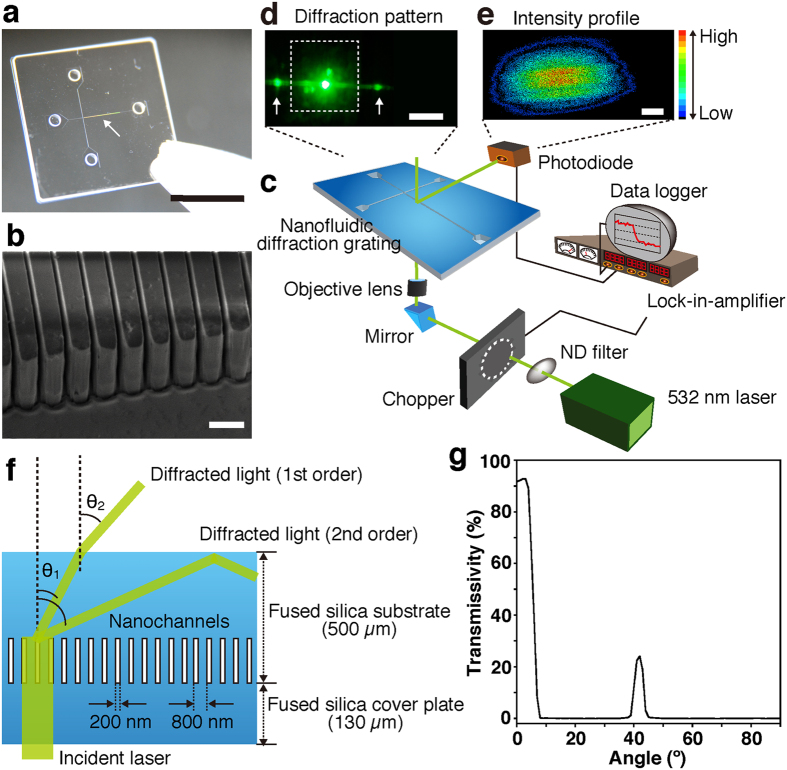
Label-free detection system using the nanofluidic diffraction grating. (**a**) A photo of the nanofluidic diffraction grating; scale bar, 1 cm. Interference fringes indicated the nanochannels were embedded in the microchannel as marked by the white arrow. (**b**) A SEM image of the nanochannels, which was 800 nm period, and 200 nm width and 2.7 μm depth fused silica nanogrooves; scale bar, 1 μm. (**c**) A schematic illustration showing the setup for label-free detection using signal changes of the diffracted light when the nanofluidic diffraction grating was filled with a sample (various liquids, DNA molecules, or amplified DNA molecules). (**d**) A photo showing the diffracted light (white arrows) and the origin of the beam; scale bar, 1 cm. The white dotted lines outline the nanofluidic diffraction grating. (**e**) A two-dimensional (2D) intensity profile of the diffracted light; scale bar, 1 mm. Color gradation showing intensity variation; red and black colors mean high and low intensities, respectively. (**f**) A schematic illustration showing the diffraction of light through the device. (**g**) Transmissivity for the wavelength of 532 nm at each output angle. The 532 nm laser beam has some transmittance at 42°.

**Figure 2:**
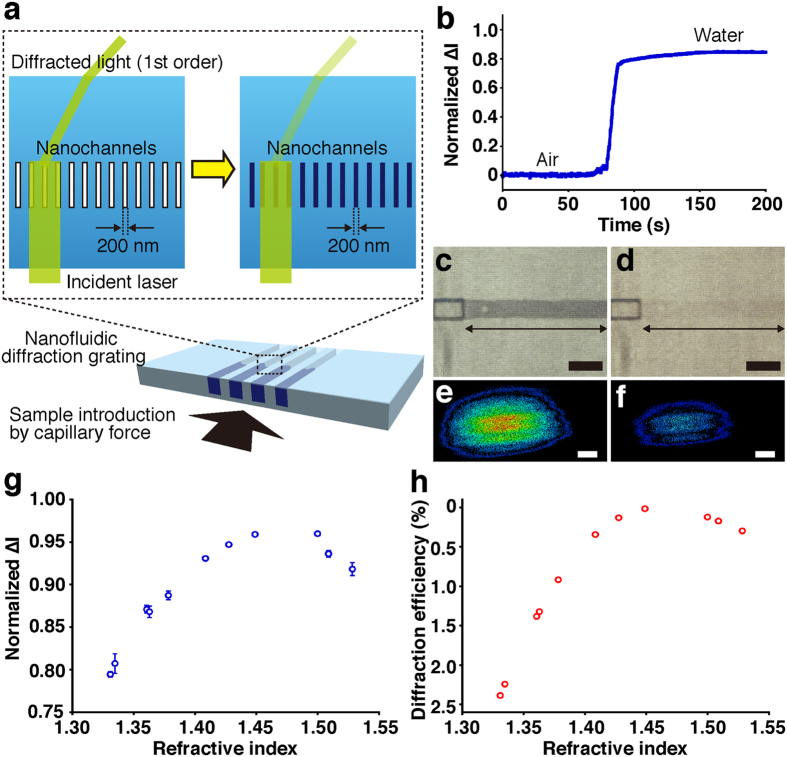
Label-free detection of various liquids introduced into the nanofluidic diffraction grating. (**a**) A schematic illustration showing introduction of various liquids into the nanochannels by capillary force. (**b**) Time-course monitoring of normalized ∆I of the diffracted light during water introduction. Normalized ∆I was defined as the intensity difference from the initial state after sample introduction. (**c**) A micrograph of the nanochannels as indicated by the double-headed arrow before water introduction; scale bar, 50 μm. (**d**) A micrograph of the nanochannels as indicated by the double-headed arrow after water introduction; scale bar, 50 μm. (**e**) Intensity profile of the diffracted light before water introduction; scale bar, 1 mm. (**f**) Intensity profile of the diffracted light after water introduction; scale bar, 1 mm. (**g**) Normalized ∆I plot derived from signal changes by the transit of various liquids, such as methanol, water, acetone, ethanol, isopropanol, tetrahydrofuran, cyclohexane, chloroform, toluene, *o-*xylene, and chlorobenzene, through the nanochannels, with respect to the refractive indices of them. Error bars show the standard deviation for a series of measurements (N = 5). (**h**) Diffraction efficiency derived from RCWA versus the refractive indices of the various liquids. The diffraction efficiency calculated by the RCWA simulation showed the percentage for an allocated intensity of the diffracted light of the first order based on the intensity of the incident laser beam.

**Figure 3:**
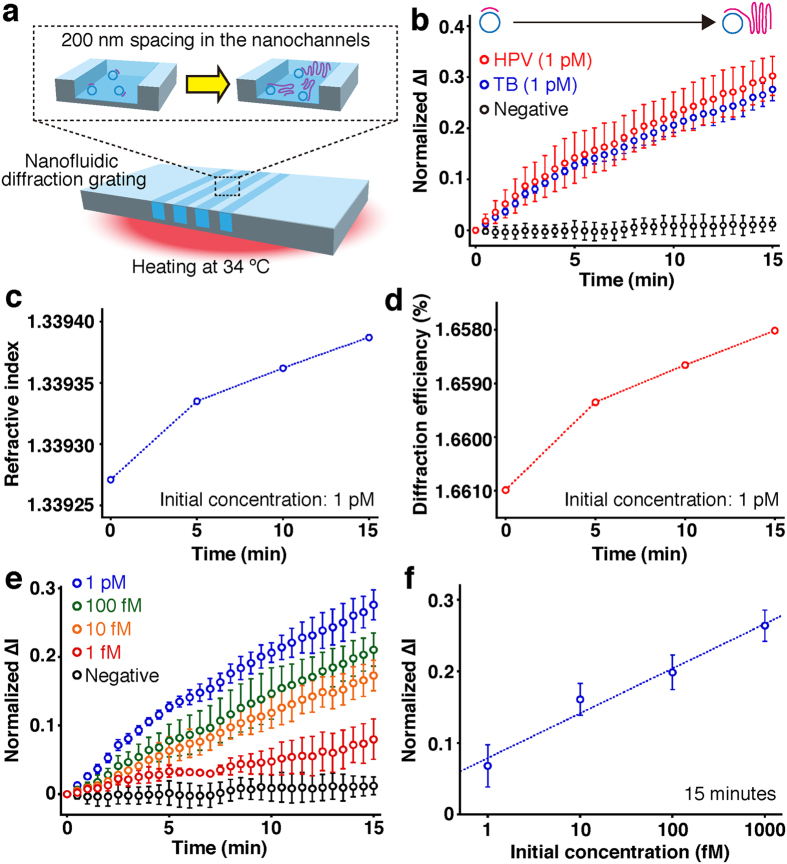
Label-free detection of real-time DNA amplification in the nanofluidic diffraction grating. (**a**) A schematic illustration showing real-time DNA amplification in the device at 34 °C. (**b**) Time-course monitoring of normalized ∆I of the diffracted light during real-time DNA amplification with different target sequences. Target sequences of amplified DNA molecules were human papillomavirus (HPV, red circles, initial concentration: 1 pM) sequence and *tubercle bacilli* (TB, blue circles, initial concentration: 1 pM) sequence. Negative control data (black circles) were obtained with no target sequence. Error bars show the standard deviation for a series of measurements (N = 3). (**c**) Refractive index versus amplification time. The initial concentration of TB sequence was 1 pM. (**d**) Diffraction efficiency derived from RCWA versus amplification time. The initial concentration of TB sequence was 1 pM. (**e**) Time-course monitoring of normalized ∆I of the diffracted light during real-time DNA amplification for TB sequence with different initial concentrations. Negative control data (black circles) were obtained with no target sequence. Error bars show the standard deviation for a series of measurements (N = 3). (**f**) Normalized ∆I plot derived from signal changes by DNA amplification for 15 min in the nanochannels, with respect to the initial concentration of TB sequence. Error bars show the standard deviation for a series of measurements (N = 3).
