# Supplementary material for: Label-free detection of real-time DNA amplification using a nanofluidic diffraction grating
Source: Sci Rep. 2016 Aug 17;6:31642. doi: 10.1038/srep31642 (PMC4987677; doi:10.1038/srep31642)
Supplement: Supplementary Information [file srep31642-s1.doc]

*Supplementary information for*

Label-free detection of real-time DNA amplification using a nanofluidic diffraction grating

*Takao Yasui^1,2,3^*, Kensuke Ogawa^1^, Noritada Kaji^1,2^, Mats Nilsson^4^, Taiga Ajiri^5^, Manabu Tokeshi^2,6^*, Yasuhiro Horiike^7^, and Yoshinobu Baba^1,2,8^**

^1^Department of Applied Chemistry, Graduate School of Engineering, Nagoya University, Furo-cho, Chikusa-ku, Nagoya 464-8603, Japan

^2^ImPACT Research Center for Advanced Nanobiodevices, Nagoya University, Furo-cho, Chikusa-ku, Nagoya 464-8603, Japan

^3^JST, PRESTO, Graduate School of Engineering, Nagoya University, Furo-cho, Chikusa-ku, Nagoya 464-8603, Japan

^4^Science for Life Laboratory, Department of Biochemistry and Biophysics, Stockholm University, Se-171 21 Solna, Sweden

^5^Graduate School of Chemical Sciences and Engineering, Hokkaido University, Sapporo 060-8628, Japan

^6^Division of Applied Chemistry, Faculty of Engineering, Hokkaido University, Sapporo 060-8628, Japan

^7^National Institute for Materials Science, Tsukuba 305-0044, Japan,

^8^Health Research Institute, National Institute of Advanced Industrial Science and Technology (AIST), Takamatsu 761-0395, Japan.

*Corresponding authors: (T. Yasui) Phone: +81-52-789-4611; Fax: +81-52-789-4666; E-mail: yasui@apchem.nagoya-u.ac.jp; (M. Tokeshi) Phone: +81-11-706-6744; Fax: +81-11-706-6745; E-mail: tokeshi@eng.hokudai.ac.jp; (Y. Baba) Phone: +81-52-789-4664; Fax: +81-52-789-4666; E-mail: babaymtt@apchem.nagoya-u.ac.jp

**This file includes:**

Materials and Methods

Supplementary Figures 1-8

Supplementary Tables 1-4

Supplementary references

**Materials and Methods**

**Fabrication procedure for nanochannels embedded in a microchannel.** The nanochannels embedded in the microchannel were fabricated on fused silica substrates (Covalent Materials Corp.). First a 10 nm thick Cr layer and a 10 nm thick Pt layer were deposited on the substrate by sputtering as drawn in Fig. S1a. Positive resist (ZEP-520A, Zeon Corp.) was coated on the Cr/Pt layers by spin-coating (Fig. S1b), and then the nanochannels pattern was drawn by electron beam (EB) lithography (ELS-7500, Elionix Inc.) in Fig. S1c. Ni electroplating onto the nanochannels pattern in the resist was employed (Fig. S1d); the Ni was a mask for a reactive ion etching process to fabricate the nanochannels. After removal of the resist (Fig. S1e), positive photoresist (OFPR8600, Tokyo Ohka Kogyo Co., Ltd.) was spin-coated on the Ni mask patterned substrate (Fig. S1f), and then the microchannel pattern with a width of 25 µm was formed by photolithography (Fig. S1g). The nanochannels embedded in the microchannel was formed by neutral loop discharge (NLD) plasma etching under CF_4_ gas ambient (Fig. S1h). Then the resist, Ni mask, and the Cr/Pt metal layers were removed from the substrate using a sulfuric acid and heating at 180 ºC for 2 hours, using a mixture of sulfuric acid and hydrogen peroxide (4:1 by weight percent) and heating at 180 ºC for 2 hours, using a mixture of nitric acid and hydrochloric acid (3:1 by weight percent) and heating at 180 ºC for 30 minutes, and using a Cr etchant (H_2_O/Ce(NH_4_)_2_-(NO_3_)_6_/HClO_4_, 85:10:5 by weight percent), followed by 1% hydrofluoric acid for 5 seconds. The acidic cleaning solution and the Cr etchant were discarded after use of them. Reservoir via holes of 1.5 mm diameter for the microchannel were formed with an ultrasonic driller (SOM-121, Shinoda Co., Ltd.). Before sealing with a cover plate, the patterned substrate was cleaned by using a mixture of sulfuric acid and hydrogen peroxide (4:1 by weight percent) and heating at 180 ºC for 2 hours, followed by a mixture of NH_3_:H_2_O_2_:H_2_O (2:1:7 by weight percent) and heating at 180 ºC for 30 minutes (Fig. S1i). The mixtures were discarded after use of them. The patterned substrate was sealed using a 130 µm thick fused silica cover plate (Covalent Materials Corp.) by dipping both of them into H_2_SiF_6_ and bonding them at 5 MPa and 65 ºC for 12 hours (Fig. S1j).


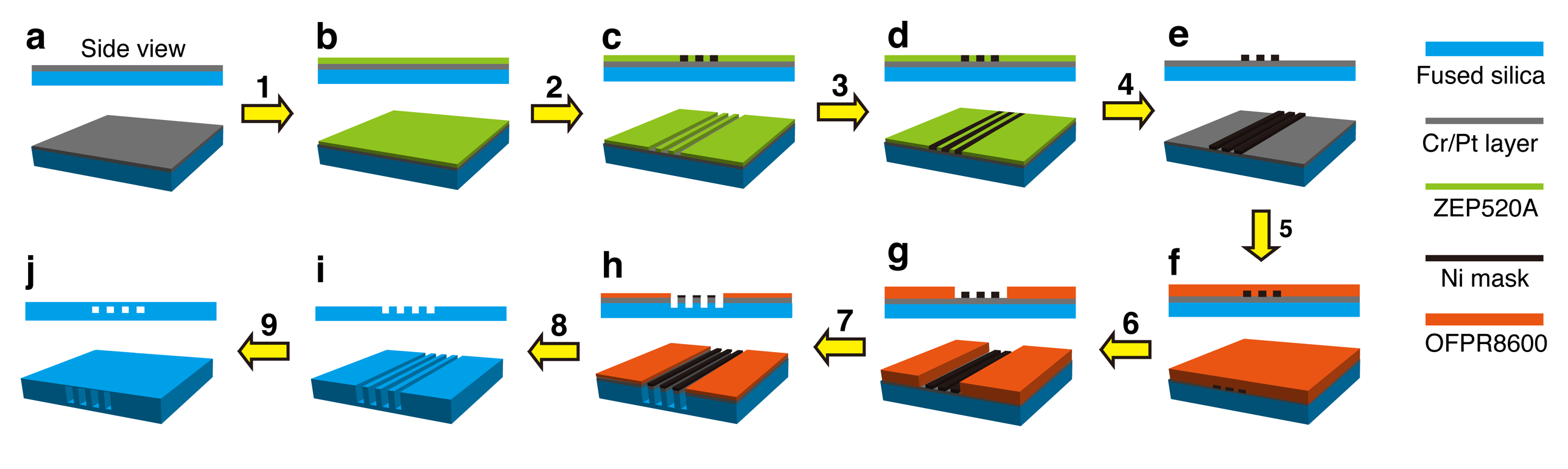


**Figure S1 Schematic of the fabrication procedure for nanochannels embedded in a microchannel.** The nanochannels embedded in the microchannel is formed on a fused silica substrate by utilizing lithographic techniques. Components include fused silica substrate (cyan), Cr/Pt metal layers (grey), electron beam resist (green), nickel mask (black), and photoresist (orange).

**Calculation of refractive index for various liquids at arbitrary wavelengths.** All various liquids were purchased from Wako Pure Chemical Industries, Ltd. Refractive indices of various liquids were measured with a high-precision refractometer (KPR-2000, Shimadzu Corp.,) at room temperature with 50% humidity. The refractive index at 532 nm wavelength was calculated using the five-term Herzberger equation (1):
*n(λ) = A + Bλ^2^ + Cλ^4^ + D/(λ^2^ – 0.028) + E/(λ^2^ – 0.028)^2^* (1),
where *A*, *B*, *C*, *D*, and *E* are approximation coefficients, *n*(*λ*) is an refractive index at an arbitrary wavelength, and *λ* is the arbitrary wavelength. *A*, *B*, *C*, *D*, and *E* are solutions to a system of equations from five combinations of wavelength and refractive index. The measured refractive indices at five different arbitrary wavelengths (587.6, 656.3, 486.1, 546.1, and 435.8 nm) are summarized in Table S1 along with the respective calculated indices at 532 nm. The refractive index of fused silica at 532 nm is 1.46071[^1^](#_ENREF_1).

**Table S1 Refractive indices of various liquids** at several wavelengths.

|  | Refractive index | | | | | |
| --- | --- | --- | --- | --- | --- | --- |
| various liquids | Fraunhofer lines | | | | | 532 nm |
|  | d line  (587.6 nm) | C line  (656.3 nm) | F line  (486.1 nm) | e line  (546.1 nm) | g line  (435.8 nm) |  |
| Methanol | 1.32916 | 1.32754 | 1.33293 | 1.33049 | 1.33582 | 1.33098 |
| Water | 1.33281 | 1.33094 | 1.33688 | 1.33423 | 1.33995 | 1.33480 |
| Acetone | 1.35851 | 1.35658 | 1.36320 | 1.36012 | 1.36693 | 1.36073 |
| Ethanol | 1.36086 | 1.35902 | 1.36508 | 1.36227 | 1.36836 | 1.36286 |
| Isopropanol | 1.37612 | 1.37419 | 1.38060 | 1.37767 | 1.38415 | 1.37826 |
| Tetrahydrofuran | 1.40622 | 1.40419 | 1.41103 | 1.40784 | 1.41481 | 1.40850 |
| Cyclohexane | 1.42506 | 1.42286 | 1.43031 | 1.42683 | 1.43444 | 1.42754 |
| Chloroform |  | | | | | 1.449* |
| Toluene |  | | | | | 1.50* |
| *o*-xylene | 1.50365 | 1.49910 | 1.51490 | 1.50738 | 1.52429 | 1.50889 |
| Chlorobenzene | 1.52272 | 1.51779 | 1.53487 | 1.52672 | 1.54497 | 1.52837 |

*From Ref. [2](#_ENREF_2)

**Label-free detection of molecular composition exchange by electroosmotic flow.** The label-free detection system based on the change of the refractive index could also recognize different molecular compositions in solution. We exchanged solutions by electroosmotic flow in Fig. S2a; *e.g.*, from water to buffer (3×TBE; 267 mM tris-borate and 6 mM EDTA in water) (Fig. S2b) or from buffer (3×TBE) to buffer (1×TE; 10 mM tris and 1 mM EDTA in water) (Fig. S2c). Normalized ∆I (∆I was defined as the intensity difference from the initial state after sample introduction) was calculated by subtracting the average value of normalized intensity for 10 s from the normalized intensity at each point (Figs. S2b and S2c). The normalized ∆I at each molecular composition exchange was plotted in Fig. S2d.


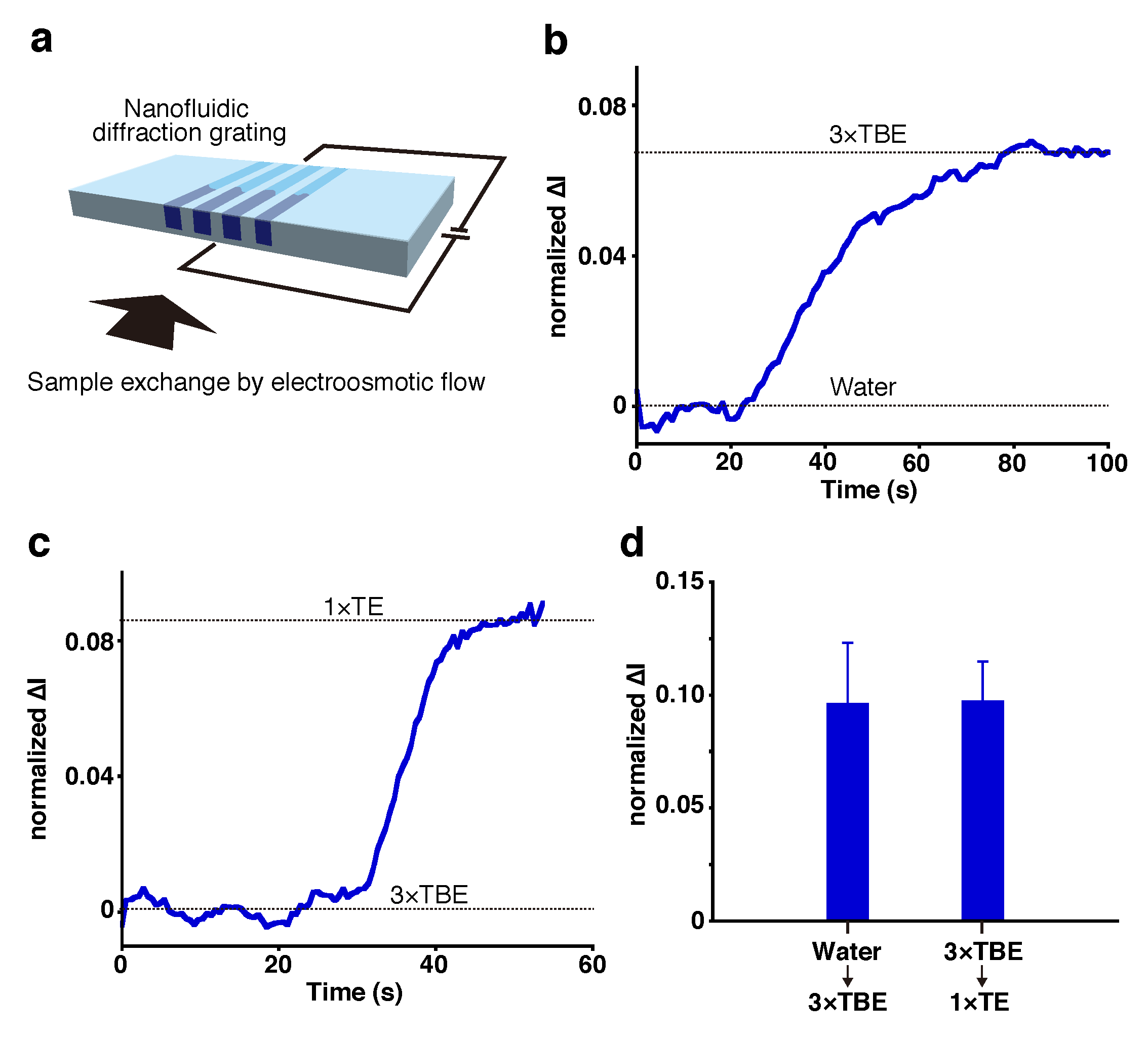


**Figure S2 Label-free detection of molecular composition exchange by electroosmotic flow.** (a) A schematic illustration showing exchange of molecules inside the nanochannels by electroosmotic flow. (b) Time-course monitoring of smoothed ∆I of the diffracted laser beam during solvent exchange from water to buffer (3×TBE; 267 mM tris-borate and 6 mM EDTA in water). (c) Time-course monitoring of smoothed ∆I of the diffracted laser beam during solvent exchange from one buffer (3×TBE) to another buffer (1×TE; 10 mM tris and 1 mM EDTA in water). (d) ∆I plots at each molecular composition exchange. Error bars show the standard deviation for a series of measurements (N = 5).

**Label-free detection of molecular composition exchange by electroosmotic flow.** The normalized intensity change of the diffracted light when introducing DNA molecules into the 200 nm wide grooves in the nanofluidic diffraction grating showed that our label-free detection system could achieve good sensitivity for label-free detection of DNA molecules (Fig. S3). We monitored the normalized intensity change, normalized ∆I, of the diffracted light when DNA molecules were electrophoretically passed through the device (Fig. S3a); the normalized ∆I changed from its initial state (only buffer) value to its terminal state (DNA molecules in buffer) value. Figures S3b and S3c show results for the label-free detection of DNA molecules as normalized ∆I versus DNA length (DNA concentration: 1.52 µM) and normalized ∆I versus concentration (DNA length: 200 bp), respectively. Both had linear relationships between normalized ∆I and DNA length from 100 to 500 bp, and between normalized ∆I and DNA concentration from 10 to 300 ng/µL.

Since the normalized intensity change of the diffracted light when introducing DNA molecules having no absorbance at 532 nm into the 200 nm wide grooves in the nanofluidic diffraction grating showed a linear relationship between normalized ∆I and DNA length or concentration, the changes of refractive index for introduction of DNA molecules contributed to normalized ∆I. We measured refractive indices of 200 bp DNA molecules in response to their concentration with the high-precision refractometer, and the values are summarized in Supplementary Table S2. It was clear that the refractive index of 200 bp DNA molecules changed with the DNA concentration (Fig. S3d). Using those refractive indices and the RCWA method, we simulated diffraction efficiency for each DNA concentration, and it had good linearity with the DNA concentration from 50 to 200 ng/µL as shown in Fig. S3e. A decrease of diffraction efficiency in response to concentration of 200 bp DNA molecules occurred with an accompanying intensity decrease of diffracted light, resulting in an increase of normalized ∆I. Due to the fact that the high-precision refractometer could not recognize the difference for the refractive indices between DNA molecules below 50 ng/µL and buffer solution (0 ng/µL DNA concentration), we could not identify any differences in diffraction efficiency below 50 ng/µL. But unexpectedly, our label-free detection system could resolve the signal changes below 50 ng/µL DNA concentration, while resolution of the high-precision refractometer in the bulk was limited to 100 ng/µL. In terms of resolution, our label-free system is superior to the conventional high-precision refractometer.

**
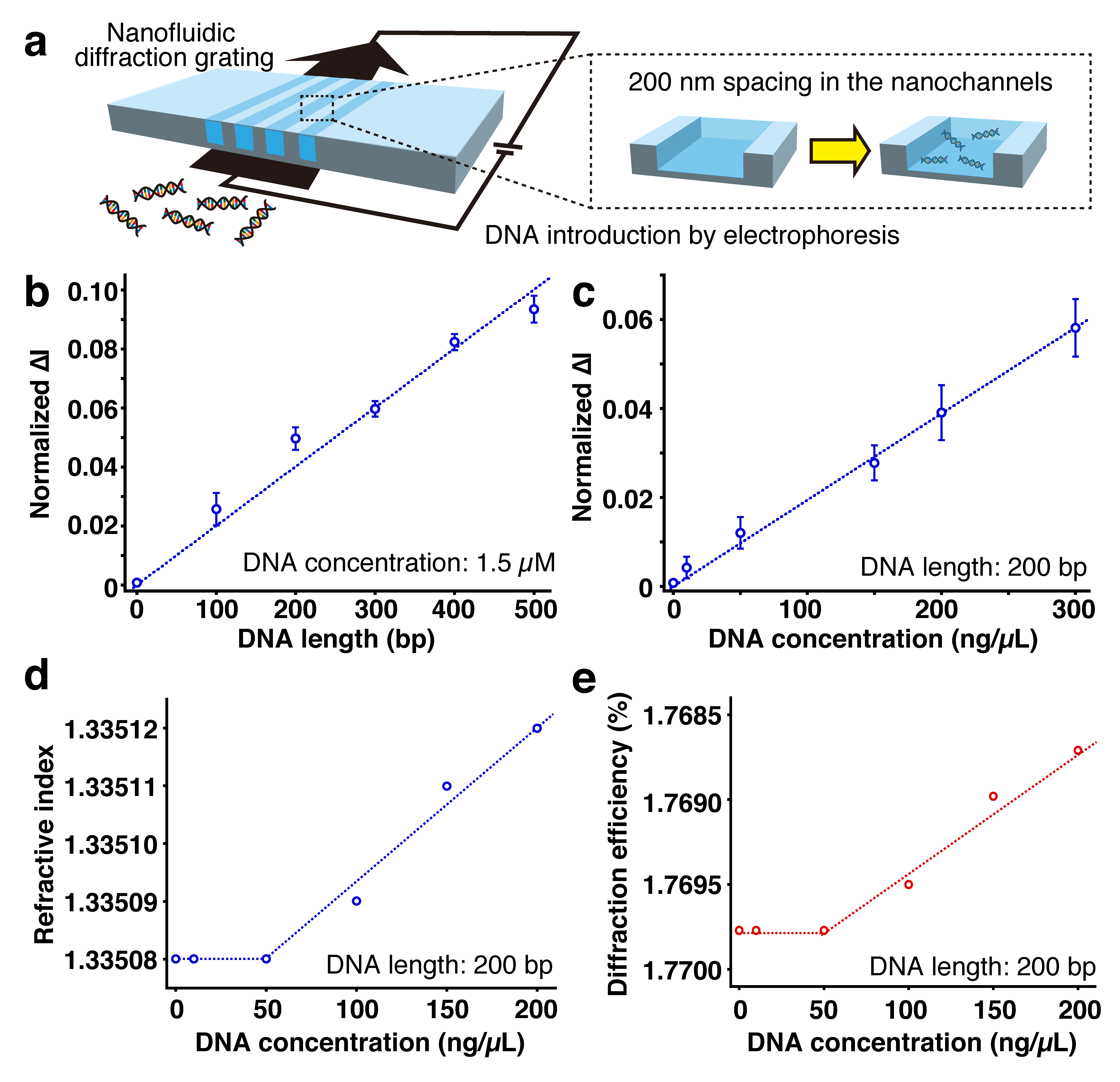
**

**Figure S3 Label-free detection of DNA molecules introduced into the nanofluidic diffraction grating.** (a) A schematic illustration showing introduction of DNA molecules into the device by electrophoresis. (b) Normalized ∆I plot derived from signal changes by transit of DNA molecules through the device, with respect to DNA length. Concentrations of each DNA length were 1.52 µM. There was a linear relationship between normalized ∆I and DNA length and it ranged from 100 to 500 bp (blue dotted line). Error bars show the standard deviation for a series of measurements (N = 5). (c) Normalized ∆I plot derived from signal changes by transit of DNA molecules through the device, with respect to concentration of 200 bp DNA molecules. Normalized ∆I – DNA concentrations showed a linear relationship as indicated by the blue dotted line. Error bars show the standard deviation for a series of measurements (N = 10). (d) Refractive index versus concentration of 200 bp DNA molecules. (e) Diffraction efficiency derived from RCWA versus concentration of 200 bp DNA molecules.

**Effect of light absorption on label-free detection**. Coupled with the label-free detection of DNA molecules (non-absorbing molecules), we considered the effect of light absorption by target molecules on label-free detection at 532 nm. In the case of absorbing molecules, we must deal with label-free results based on not only refractive index but also thermal energy. We monitored the signal changes for introduction of sunset yellow FCF (Wako Pure Chemical Industries, Ltd.), which showed some absorbance at 532 nm and transferred absorbed energy to heat (thermal) energy, in the concentration range from 100 nM to 10 mM (Fig. S4). Sunset yellow FCF molecules were dissolved in 3×TBE buffer, which consisted of 267 mmol/L Tris-borate, 6 mmol/L EDTA, and had pH of 8.3. Signal intensities in 3×TBE were normalized to 1.0. Normalized ∆I was calculated by subtracting the average value of normalized intensity for 10 s from the normalized intensity at each point. The data for sunset yellow FCF showed a linear relationship between normalized ∆I and logarithmic scale concentration in the range from 100 nM to 10 mM, and the LOD was 100 nM, which gave a signal at 3 SDs (standard deviations) above the background. As described earlier, when sunset yellow FCF was excited by light, absorbed energy was changed into thermal energy, and then this thermal energy caused the solution temperature to rise. Finally this temperature rise could change the refractive index of the solvent or fused silica substrate, and therefore sunset yellow FCF molecules had two types of refractive index change; in one, the refractive index of the samples themselves change, and in the other, the refractive index is changed by the temperature rise of the solution. By measuring signal fluctuations of absorbing molecules, which were attributed to both changes of the refractive index and thermal energy, we saw that the calibration curve for a semi-logarithmic plot might result from these multiple factors.

**Valid volume of label-free detection.** The radius of the laser beam spot, *r*, in the nanofluidic diffraction grating was calculated to be 1081 nm using *r = 061λ/NA*, where *NA* is the numerical aperture of the objective lens. Within the beam diameter of 2162 nm there were two or three periodic nanogrooves (800 nm period and 200 nm wide grooves), and then the 200 nm wide grooves in the nanochannels, through which samples could pass, should have an area range of 8.02×10^5^ ≤ S ≤ 1.01×10^6^ (nm^2^). Because the nanochannels had the height of 2.7 µm, the valid volume of label-free detection was roughly estimated to be in the range of 2.17×10^9^ ≤ V ≤ 2.73×10^9^ (nm^3^), or around 2 fL.


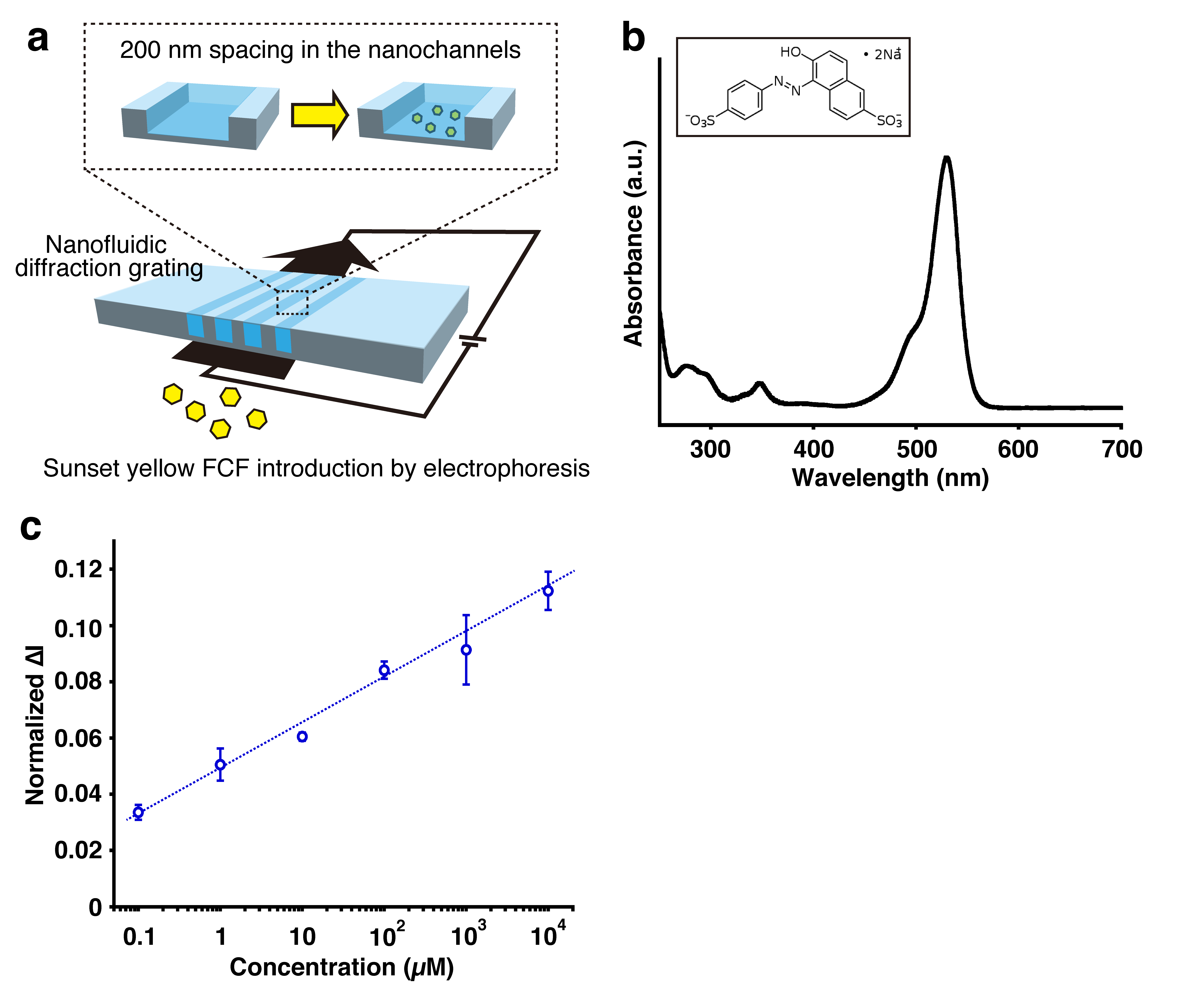


**Figure S4 Label-free detection of sunset yellow FCF molecules introduced into the nanofluidic diffraction grating.** (a) A schematic illustration showing sunset yellow FCF molecules introduced into the nanochannels by electrophoresis. (b) Absorption spectrum of sunset yellow FCF molecules. The inset shows the chemical structural formula of sunset yellow FCF. (c) Time-course monitoring of smoothed normalized ∆I of the diffracted laser beam during introduction of sunset yellow FCF in a wide range of concentrations from 100 nM to 10 mM. (d) Normalized ∆I vs. concentration plot derived from signal changes by introduction of sunset yellow FCF molecules into the nanochannels. Error bars show the standard deviation for a series of measurements (N = 3). The blue dotted line shows a linear relationship between normalized ∆I and the logarithmic scale concentrations which range from 100 nM to 10 mM.

**Calculation of refractive index for the concentration of DNA molecules at arbitrary wavelengths.** Refractive indices of DNA molecules were measured with the high-precision refractometer (KPR-2000) at room temperature and 50% humidity. The refractive index at 532 nm wavelength was calculated using the five-term Herzberger equation (1). The measured refractive indices at five different arbitrary wavelengths (587.6, 656.3, 486.1, 546.1, and 435.8 nm) are summarized in Table S2 along with the respective calculated indices at 532 nm.

**Table S2 Refractive indices of DNA molecules at several wavelengths.**

|  | Refractive index | | | | | |
| --- | --- | --- | --- | --- | --- | --- |
| Concentration of DNA molecules (ng/µL) | Fraunhofer lines | | | | | 532 nm |
|  | d line  (587.6 nm) | C line  (656.3 nm) | F line  (486.1 nm) | e line  (546.1 nm) | g line  (435.8 nm) |  |
| 0* | 1.33312 | 1.33124 | 1.33719 | 1.33453 | 1.34026 | 1.33508 |
| 10 | 1.33312 | 1.33123 | 1.33719 | 1.33452 | 1.34025 | 1.33508 |
| 50 | 1.33311 | 1.33123 | 1.33719 | 1.33452 | 1.34025 | 1.33508 |
| 100 | 1.33313 | 1.33125 | 1.33720 | 1.33454 | 1.34026 | 1.33509 |
| 150 | 1.33314 | 1.33126 | 1.33722 | 1.33455 | 1.34028 | 1.33511 |
| 200 | 1.33315 | 1.33127 | 1.33723 | 1.33457 | 1.34029 | 1.33512 |

*0 ng/µL DNA concentration means only buffer solution (1×TE: 10 mmol/L Tris-HCl, 1mmol/L EDTA; pH = 8.0).

**Circle-to-circle amplification (C2CA).** As shown in Fig. S5, the whole process, using a padlock probe for a specific sequence, amplifies DNA strands specifically to large numbers of oligonucleotides. The C2CA scheme consists of 5 steps: hybridization & ligation; coupling; the 1st rolling circle amplification (RCA); digestion; and ligation & the 2nd RCA. In the hybridization & ligation step, target sequences (*tubercle bacilli*: TB or human papillomavirus: HPV, summarized in Table S3) were incubated for 5 min at 60 ºC in the solution containing 100 nM phosphorylated padlock probes, 50 nM capture oligonucleotides, 0.2 µg/µL purified BSA (BioLabs), 1x ampligase buffer (Epicentre), and 250 mU/µL ampligase (Epicentre). After hybridization & ligation, to capture the target sequence in the solid phase, the ligation mixture was coupled for 5 min at room temperature with 2 mg/mL Dynabeads® MyOne™ Streptavidin T1 (Invitrogen), which had been previously washed three times with washing buffer including 40 mM Tris-HCl (Sigma) (pH 7.5), 20 mM EDTA (Sigma), 0.4 % tween-20 (Sigma), and 0.4 M NaCl (Sigma). The capture oligonucleotides with biotin modification at the 5’ end hybridize to the target sequence, resulting in immobilization of the target sequence onto magnetic beads via biotin-streptavidin bonding. After coupling the ligation mixture with the magnetic beads, the magnetic beads were washed with the washing buffer once.

For the 1st RCA reaction, the magnetic beads were dispersed in 0.2 µg/µL BSA, 125 µM dNTP (Fermentas), 1×phi29 DNA polymerase buffer (Fermentas), and 100 mU/µL phi29 DNA polymerase (Fermentas) following the removal of the washing buffer, and then the dispersed solution was incubated for 20 min at 37 ºC and then inactivated for 1 min at 65 ºC. The amplified products were restriction-digested in the next digestion step. 5 µL of reaction mixture, which had a composition of 0.2 µg/µL BSA, 1×phi29 DNA polymerase buffer, 40 mU/µL AluⅠ (BioLabs), and 120 nM replication oligonucleotide, was added to 20 µL of the 1st RCA solution. The digestion was done for 1 min at 37 ºC. After inactivation of the restriction enzyme for 1 min at 65 ºC, the magnetic beads were discarded. Lastly, the ligation & 2nd RCA step was initiated by adding 25 µL of digested solution to 25 µL of reaction solution. The reaction solution for the ligation & 2nd RCA contained 0.2 µg/µL BSA, 0.67 mM ATP, 14 mU/µL T4 DNA ligase (Fermentas), 1×phi29 DNA polymerase buffer, 100 µM dNTP, and 60 mU/µL phi29 DNA polymerase. The ligation & 2nd RCA time was 20 min at 34 ºC. To avoid unexpected amplification, all preparations were done at 4 ºC.





**Figure S5 Structures of oligonucleotides used in this study.** Schematic of hybridization & ligation, coupling, the 1st rolling circle amplification (RCA), digestion, and ligation & the 2nd RCA. The structures of the target sequence, padlock probe, capture oligonucleotide, and replication oligonucleotide are shown in black, blue, red, and blue, respectively. The 5’ end of the capture oligonucleotide has biotin and connects to magnetic beads with streptavidin coating. Padlock probe is ligated after hybridization to the target sequence. The initiation of the 1st RCA reaction is followed by ligation of the padlock probe. The resulting product is composed of repeated sequences of the padlock probe. This repeated product is restriction-digested via the replication oligonucleotide, monomerized, and then circularized. These circles are subsequently used as templates for the 2nd RCA.

**Oligonucleotides.** The DNA sequences of target sequence for *tubercle bacilli* (TB), target sequence for human papillomavirus (HPV), padlock probe for TB, padlock probe for HPV, capture oligonucleotide for TB, capture oligonucleotide for HPV, and replication oligonucleotide are summarized in Table S3. These oligonucleotides were purchased from Integrated DNA Technologies. The purification grade of the padlock probes was PAGE one and that of all others was HPLC purification. The padlock probes were phosphorylated before use in the RCA. 1 µM padlock probes were incubated in 1 mM ATP (Fermentas), 0.1 U/µl T4 PNK polynucleotide Kinase (Fermentas), and 1x reaction buffer A PNK (Fermentas) for 30 min at 37 ºC, followed by a second incubation for 20 min at 65 ºC.

**Table S3 Oligonucleotide sequences.**

| Name | Oligonucleotide sequences (5’ → 3’) |
| --- | --- |
| Target sequence for *tubercle bacilli* (TB) | CGTTAGCAATCACCCTGCTCTGTGAAGTCCGGACTTTCCTCGACTCGACGCTGAACCTCGTGAATCCACACAAGCCCTACG |
| Target sequence for human papillomavirus (HPV) | GAGGAGGATGAAATAGATGGTCCAGCTGGACAAGCAGAACCGGACAGAGCCCATTACAATATTGTAACCTTTTGTTGCAAGTGTGACTC |
| Padlock probe for TB | GATTCACGAGGTTCAGGTGCGACACATGACATCAACGTGTATGCAGCTCCTCAGTACGTAGGGCTTGTGTG |
| Padlock probe for HPV | ACAAAAGGTTACAATATTGCAGCTTGATTCCGAGATGTGCAGTGTATGCAGCTCCTCAGTAGAGTCACACTTGCA |
| Capture oligonucleotide for TB | Biotin-CTCTCTCTCTAGGAAAGTCCGGACTTCACAGAGCAGGGTGATTGCTAACG |
| Capture oligonucleotide for HPV | Biotin-CTCTCTCTCTGTTCTGCTTGTCCAGCTGGACCATCTATTTCATCCTCCTC |
| Replication oligonucleotide | GTGTATGCAGCTCCTCAGTA |

**Temperature dependence of an activity for phi29 DNA polymerase.** Phi29 DNA polymerase can be used to amplify DNA molecules in the RCA reaction. Phi29 DNA polymerase is known to be highly processive in the absence of any accessory proteins and strand displacement activity[^5^](#_ENREF_5). The RCA occurs in a linear fashion and the amplification efficiency depends on activity of phi29 DNA polymerase. Previously, it was shown that the highly processive phi29 DNA polymerase can synthesize a DNA strand at the rate of at least 1000 nt/min[^6^](#_ENREF_6). This phi29 DNA polymerase-catalyzed RCA reaction proceeds with an estimated half-life of 11 hours at 37 ºC, yielding ~0.5 Mb of DNA strands per probe. To the best of our knowledge there are no detailed reports about the temperature dependency of the activity of phi29 DNA polymerase in an RCA reaction. In Fig. S6, we report the activity of phi29 DNA polymerase at different temperatures for 30 min, and determine the proper temperature to amplify 1 pM target sequence for TB. After coupling the ligation mixture with the magnetic beads, the magnetic beads were washed with the washing buffer once.

We changed the temperature for the 1st RCA and quantitatively investigated the temperature dependence on the RCA reaction. Changing the 1st RCA temperature should dramatically influence the length of the 1st RCA product and the number of templates for the 2nd RCA. After the 2nd RCA reaction, we labelled the 2nd RCA products using the following step, and counted the number of blobs for the 2nd RCA products using the amplified single-molecule detection technique (Fig. S6a)[^7^](#_ENREF_7). 50 µL of reacted solution with the 2nd RCA products was labelled in 50 µL of 0.5 M EDTA, 1 M Tris-HCl (pH 8.0), 10 % tween-20, 5 M NaCl, and 1 µM detection oligonucleotide (5’ Alexa Fluor 555-GTTGATGTCATGTGTCGCAC 3’) for 2 min at 70 ºC and followed by further reaction for 15 min at 55 ºC. To investigate non-specific amplification, we carried out a negative control simultaneously with all experiments; the negative control meant the RCA reaction without target sequence. Generally, the number of blobs resulting from a negative control is around 100, most of our results were around 100, and therefore we concluded that our RCA reaction had non-specific amplification.

We present the temperature dependence of phi29 DNA polymerase on the 1st RCA in Fig. S6b. Because all experimental conditions except for the 1st RCA temperature were completely the same, the number of blobs in Fig. S6b should be affected by the 1st RCA temperature. Up to 34 ºC the number of blobs increased and there was a linear relationship between the number of blobs and temperature. Between 34 and 38 ºC there was a local maximum. Above 38 ºC there was a monotonic decrease and also a linear relationship between the number of blobs and temperature. The number of blobs relatively indicates how long the sequence of the padlock probe was replicated; that is, how phi29 DNA polymerase was activated in response to temperature changes. For instance the activity of phi29 DNA polymerase at 34 ºC was double that at 25 ºC. To investigate details of the time dependence on RCA reaction at 34 ºC, we plotted the results of time-course data (Fig. S6c). The number of blobs increased in response to the 1st RCA reaction time. In the same fashion as the temperature for RCA reaction, we saw changing the 1st RCA time significantly affected the length of the 1st RCA product and the number of templates for the 2nd RCA.





**Figure S6 Temperature dependence of activity for phi29 DNA polymerase.** (a) Schematic of hybridization of the detection oligonucleotide for blob counting based on fluorescence observation (1 blob in Figs. S6b and S6c means a detection oligonucleotide hybridized 2nd RCA product). (b) Temperature dependence of phi29 DNA polymerase in the 1st RCA reaction for 30 min. Each point and error bar comes from triplicate samples at 15, 20, 25, 30, 34, 38, 42, 46, and 50 ºC. (c) Time dependence of phi29 DNA polymerase in the 1st RCA at 34 ºC. Each point and error bar comes from triplicate samples for 1, 3.75, 7.5, 15, 30, and 60 min.

**Temperature of the nanofluidic diffraction grating.** Using an infrared camera (InfReC R300, Nippon Avionics Co., Ltd.), we confirmed that the surface temperature of the nanofluidic diffraction grating was uniformly heated to around 34 ºC when we set the room temperature as 34 ºC (Fig. S7). To avoid any undesired drying out process, we sealed reservoirs of the nanofluidic diffraction grating with small pieces of aluminum sealing tape for 96 well plates (Corning Inc.) (Fig. S7a). As shown in Fig. S7b, the surface temperature without the small pieces of aluminum sealing tape was uniformly warmed to around 34 ºC.


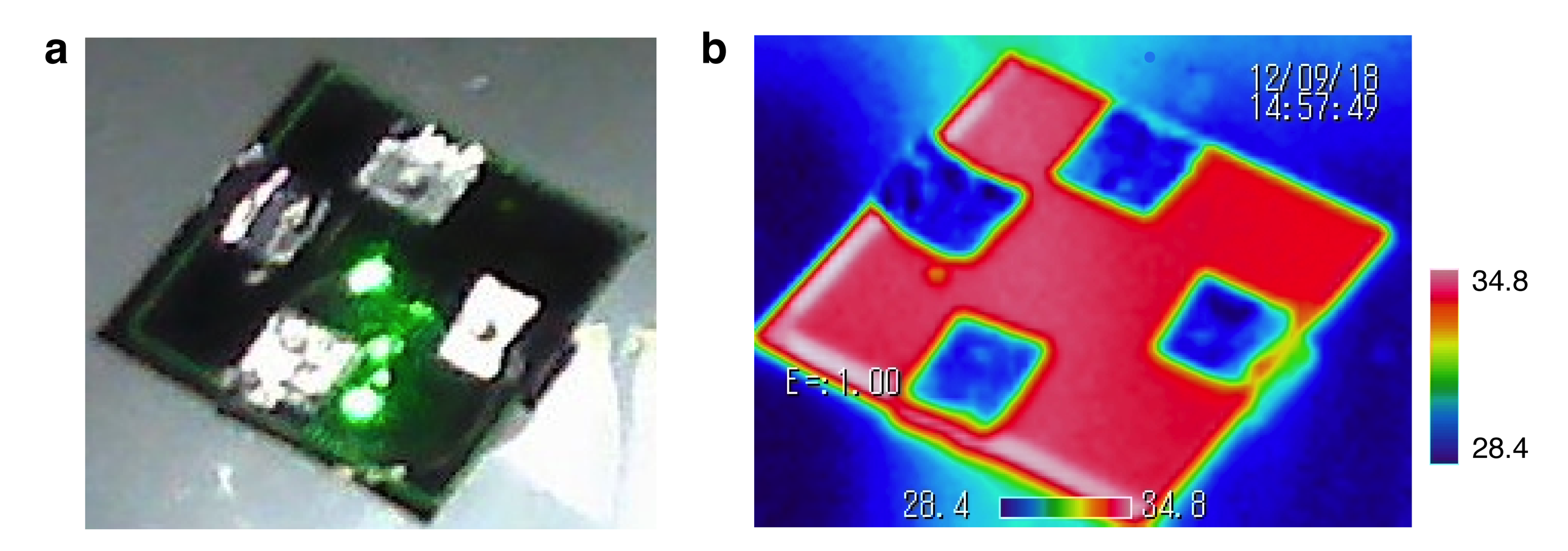


**Figure S7 Surface temperature of the nanofluidic diffraction grating.** (a) A photo of the nanofluidic diffraction grating with aluminum seals on the reservoirs. (b) Thermographic image of the nanofluidic diffraction grating with aluminum seals on the reservoirs. Color gradation shows the heat map from 34.8 to 28.4 ºC.

**Fluorescence detection of real-time DNA amplification using a molecular beacon.** We confirmed fluorescence detection of real-time DNA amplification of TB sequence using a molecular beacon (5' TET-CGACAACGTTGATGTCATGTGucg-BHQ1 3'; small letters were 2'-OMe-RNA) (Fig. S8a). The molecular beacon was added to the reaction solution for the ligation & 2nd RCA with various initial concentrations of TB sequence. In the same way as the label-free real-time DNA amplification, the ligation & 2nd RCA time was 20 min at 34 ºC (PikoReal 96 Real-Time PCR System, Thermo Fisher Scientific K.K.). During the 2nd RCA reaction, the molecular beacon opened and hybridized to the 2nd RCA products, and showed fluorescence intensity. In the way as the label-free detection and blob-counting method, fluorescence intensity increased in response to the RCA reaction time (Fig. S8b).





**Figure S8 Fluorescence detection of real-time DNA amplification using the molecular beacon.** (a) Schematic of hybridization of the molecular beacon. After hybridization of the molecular beacon to the 2nd RCA products, the molecular beacon opened and showed fluorescence intensity. (b) Time-course monitoring of fluorescence intensity during real-time DNA amplification for the TB sequence with different initial concentrations of TB sequence. Negative control data (black circles) were obtained with no target sequence. Error bars show the standard deviation for a series of measurements (N = 4). The data points of negative control, 1 pM, and 10 pM are overlapped.

**Calculation of refractive index during DNA amplification at arbitrary wavelengths.** Refractive indices of DNA molecules during the RCA reaction were measured with the high-precision refractometer (KPR-2000) as follows. After mixing 25 µL of prepared solution with 1 pM target sequence for TB processed in the digestion step and 25 µL of reaction solution for the ligation & 2nd RCA, the refractive indices were measured for 0 min. And then, we incubated the mixture at 34 ºC for 5 min, and measured the refractive indices for 5 min. We incubated the mixture at 34 ºC for another 5 min (total time: 10 min), and measured the refractive indices for 10 min. And also, we incubated the mixture at 34 ºC for another 5 min again (total time: 15 min), and measured the refractive indices for 15 min. The refractive index at 532 nm wavelength was calculated using the five-term Herzberger equation (1). The measured refractive indices at five different arbitrary wavelengths (587.6, 656.3, 486.1, 546.1, and 435.8 nm) are summarized in Table S4 along with the respective calculated indices at 532 nm.

**Table S4 Refractive indices of DNA molecules during DNA amplification at several wavelengths.**

|  | Refractive index | | | | | |
| --- | --- | --- | --- | --- | --- | --- |
| Amplification time (min) | Fraunhofer lines | | | | | 532 nm |
|  | d line  (587.6 nm) | C line  (656.3 nm) | F line  (486.1 nm) | e line  (546.1 nm) | g line  (435.8 nm) |  |
| 0 | 1.33728 | 1.33539 | 1.34142 | 1.33870 | 1.34449 | 1.33927 |
| 5 | 1.333734 | 1.33545 | 1.34147 | 1.33877 | 1.34456 | 1.33934 |
| 10 | 1.33737 | 1.33547 | 1.34150 | 1.33880 | 1.34459 | 1.33936 |
| 15 | 1.33739 | 1.33550 | 1.34153 | 1.33882 | 1.34462 | 1.33939 |

**References**

1 Malitson, I. H. Interspecimen comparison of refractive index of fused silica. *J. Opt. Soc. Am.* **55**, 1205-1208, (1965).

2 Dai, F. F., Xu, Y. H. & Chen, X. F. Enhanced and broadened SRS spectra of toluene mixed with chloroform in liquid-core fiber. *Opt. Express* **17**, 19882-19886, (2009).

3 Dean, F. B. Rapid amplification of plasmid and phage DNA using phi29 DNA polymerase and multiply-primed rolling circle amplification. *Genome Res.* **11**, 1095-1099, (2001).

4 Niel, C., Diniz-Mendes, L. & Devalle, S. Rolling-circle amplification of Torque teno virus (TTV) complete genomes from human and swine sera and identification of a novel swine TTV genogroup. *J. Gen. Virol.* **86**, 1343-1347, (2005).

5 Blanco, L. *et al.* Highly efficient DNA synthesis by the phage phi 29 DNA polymerase. Symmetrical mode of DNA replication. *J. Biol. Chem.* **264**, 8935-8940, (1989).

6 Baner, J., Nilsson, M., Mendel-Hartvig, M. & Landegren, U. Signal amplification of padlock probes by rolling circle replication. *Nucleic Acids Res.* **26**, 5073-5078, (1998).

7 Jarvius, J. *et al.* Digital quantification using amplified single-molecule detection. *Nat. Methods* **3**, 725-727, (2006).
